# Serology and magnetically controlled capsule gastroscopy for opportunistic screening of high-risk events in gastric cancer development: a cross-sectional study

**DOI:** 10.3389/fmed.2026.1874613

**Published:** 2026-08-18

**Authors:** Yuan Yang, Mao-Yao Wen, Jin-Chen Zou, Yan Huang, Xin-Zu Chen

**Affiliations:** 1 Gastric Cancer Center, Department of General Surgery, West China Hospital, Sichuan University, Chengdu, China; 2 Department of Gastrointestinal and Hernia Surgery, Second People's Hospital of Yibin City (West China Yibin Hospital, Sichuan University), Yibin, China; 3 Health Management Center, General Practice Medical Center, West China Hospital, Sichuan University, Chengdu, China; 4 Department of General Surgery, Second People's Hospital of Yibin City (West China Yibin Hospital, Sichuan University), Yibin, China; 5 Ya'an Cancer Prevention and Control Center, Ya'an People's Hospital (West China Ya'an Hospital, Sichuan University), Ya’an, China; 6 Ya'an Key Laboratory for High Altitude Medicine, Ya'an People's Hospital (West China Ya'an Hospital, Sichuan University), Ya’an, China

**Keywords:** atrophic gastritis, gastric cancer, magnetically controlled capsule gastroscopy, peptic ulcer, polyp, precancerous lesion, screening, serology

## Abstract

**Background:**

The feasibility of magnetically controlled capsule gastroscopy (MCCG) as an alternative diagnostic tool, along with a sequential serology—MCCG protocol, needs to be assessed for its effectiveness in detecting high-risk events associated with gastric cancer development.

**Methods:**

Health check-up records were collected between 2019 and 2020. Asymptomatic individuals underwent MCCG and relevant serologic examinations, which included tests for pepsinogen-I (PG-I), pepsinogen-II (PG-II), gastrin-17, carcinoembryonic antigen (CEA), and carbohydrate antigen 19-9 (CA19-9). The major outcomes from MCCG included tumors and high-risk events, specifically chronic atrophic gastritis (CAG), gastric ulcer, and gastric polyps. The detection rate of MCCG was estimated, and the predictive strength of serology for MCCG findings was analyzed.

**Results:**

A total of 1,432 eligible healthy individuals undergoing routine health check-ups were included. MCCG detected no tumors, but identified 114 cases with at least one high-risk event. The overall detection rate was 79.6 per 1,000 persons for all high-risk events with specific detection rates for CAG, ulcer, and polyps being 15.4, 14.7, and 55.2 per 1,000 persons, respectively. Older individuals showed significantly higher rates of high-risk events (*p* < 0.001), particularly among those aged 60 years and older. In the MCCG-detected CAG outcome dataset, serum levels of PG-I and the PG-I/-II ratio were significantly lower in the CAG group. Although the serologic tests did not effectively detect all high-risk events (AUC < 0.6, SEN < 15%), they exhibited excellent specificity (SPE > 90%). However, serologic CAG had a significant association with MCCG-detected CAG (adjusted diagnostic odds ratio = 10.40, 95% CI 2.08–51.98), despite demonstrating poor diagnostic strength (AUC = 0.555, SEN = 12.5%, SPE = 98.6%, PLR = 8.85, NLR = 0.89). The other serologic tests did not show significant associations or rational interpretation with MCCG findings.

**Conclusion:**

MCCG has acceptable detection rates for high-risk events associated with gastric cancer. However, as an alternative diagnostic tool, it lacks biopsy capability, which requires sequential flexible gastroscopy for suspicious lesions. Serologic CAG was significantly associated with MCCG-detected CAG and demonstrated high specificity, suggesting its potential utility in a screening algorithm. Both the screening efficacy of MCCG and the cost-effectiveness of sequential serology-MCCG protocol require further prospective investigation against an appropriate reference standard.

## Introduction

Gastric cancer is a common malignancy with heavy disease burden (1, 2). With the implementation of gastric cancer prevention and screening strategies between 1990 and 2019, a notable decline has been observed in the global incidence and mortality rates of gastric cancer (3). A declining trend in gastric cancer mortality has also been observed in China during recent decades (4). However, the proportion of early gastric cancer diagnoses currently remains at a relatively low level, estimated to be no more than 20%, which presents a substantial challenge to improving the overall survival rate of gastric cancer patients in China (5). In particular, this low proportion of early gastric cancer has correspondingly led to a high rate of disease-specific death in China (6). Therefore, the official outlines of the “Healthy China 2030” plan were issued in 2016, with the expectation of improving the 5-year overall survival rate for cancers by 15% nationwide (7). The plan covers key components including raising public awareness of cancer control and strengthening the quality control of oncological treatment (8, 9). Particularly, establishing a more powerful cancer prevention and control system was emphasized, wherein both population-based and opportunistic screening for major cancers are being promoted, including digestive system cancers (10, 11).

To improve the overall survival rates of gastric cancer at the population level, endoscopic screening remains the gold standard (12). However, resources for endoscopy are always limited to support massive screening at both national and subnational levels (13). Thus, it is important to identify and investigate high-risk events for gastric cancer to facilitate tailored prevention and screening. In particular, countries with a high incidence of gastric cancer should strive to reduce disease-specific mortality through endoscopic screening while also managing future incidence risk (14). Regarding the development of gastric cancer, Correa’s cascade from chronic gastritis, atrophic gastritis, and intestinal metaplasia, to dysplasia and gastric cancer is the classic theory of inflammatory pathway for gastric caicinogenesis (15–17). According to the guidelines by the National Health Commission of the People’s Republic of China and relevant collaborations, high-risk events for gastric cancer include chronic atrophic gastritis (CAG), gastric ulcer, gastric polyps, and other diseases (18, 19). *Helicobacter pylori* (*Hp*) infection is identified as a carcinogenetic pathogen for gastric cancer by the World Health Organization, and large-scale screening and eradication efforts are well recommended (20–22). Early detection and screening of these high-risk events can facilitate the surveillance of high-risk subpopulation, ultimately improving overall survival rates. Given these risk factors, risk stratification and prediction models have emerged as promising tools for precise gastric cancer screening (23).

The “Questionnaire-Serology-Endoscopy” triage model has been the subject of extensive research, as it enables effective stratification of subpopulations and avoids overutilization of limited endoscopic resources (11, 24). Conventional flexible gastroscopy presents challenges regarding public acceptability and screening adherence among at-risk populations to some extent (25). Moreover, magnetically controlled capsule gastroscopy (MCCG) emerges as a novel, non-invasive, and more compliant diagnostic method for screening gastric cancer and high-risk events, such as atrophic gastritis, gastric ulcer, and gastric polyps (26–28). In particular, MCCG is recommended as an alternative for those who are intolerant to conventional flexible endoscopy (19). Moreover, the serology of pepsinogens and gastrin-17 was found to be associated with gastric cancer and its high-risk events (29, 30), but they are still not recommended as independent screening techniques (19). In addition, conventional serum tumor biomarkers, such as CEA and CA19-9, have been found to have significant associations with an increased risk of gastric cancer (31, 32). Therefore, we hypothesized that (1) MCCG might exhibit acceptable detection performance, and (2) the sequential serology–MCCG protocol might effectively detect high-risk events of gastric cancer. Accordingly, a hospital-based cross-sectional study was conducted to further validate the above hypotheses under simulated scenarios of opportunistic screening.

## Methods

### Study design

This hospital-based cross-sectional study was conducted by the Sichuan Gastric Cancer Early Detection and Screening (SIGES) research group at West China Hospital, a central high-volume teaching hospital of Sichuan University. The study was conducted between May 2019 and December 2020, and was nested in a previous cross-sectional study, including 21,505 observations (33). Health check-up observations at the Health Management Center in West China Hospital were retrospectively collected and analyzed (33, 34).

### Ethics

This study retrospectively collected general information on the observations and results of tests or examinations. The SIGES serial studies were approved by the Biomedical Ethical Committee of West China Hospital, Sichuan University (identifiers: 2018-215-V1, 6 August 2018) (20, 30, 35). Informed consent was waived by the approval of the Biomedical Ethical Committee due to the retrospective nature, while personal information was anonymized throughout the entire process of data analysis and reporting.

### Eligibility and groups

Cancer-free participants who underwent health check-ups were potentially eligible if aged 18–75 years. Eligible individuals were asymptomatic and voluntarily opted for MCCG opportunistic screening. Individuals with a history of any malignancy or gastric surgery were excluded. Examinations of MCCG and relevant serologic examinations were mandatory for all included participants. All participants were evaluated for at least one high-risk event. Primary outcomes were individual MCCG events, including CAG, gastric ulcers, and gastric polyps. For secondary outcomes, participants were categorized into positive and negative groups based on the presence of any MCCG-detected event.

### MCCG technique

MCCG procedures were performed by experienced endoscopists from the Department of Gastroenterology. Given that MCCG is an opportunistic screening modality without biopsy capabilities, stratification and quality control procedures were not applicable under the OLGA/OLGIM standards (36). For the diagnosis of CAG via MCCG, the following endoscopic findings should be identified: the gastric mucosa exhibited hypopigmentation, presenting a predominantly whitish red-and-white mottled pattern; mucosal folds were flattened or even absent; submucosal vessels were partially visualized; and the mucosa showed granular or nodular changes. For the diagnosis of gastric polyps, the number, size, morphology, and location of the polyps were documented in detail. The Yamada classification system was used to categorize gastric polyps into four types based on their morphology and base characteristics: Type I, sessile with a broad base; Type II, hemispherical elevation; Type III, subpedunculated; and Type IV, pedunculated. For the diagnosis of gastric ulcers, descriptions should include the ulcer’s morphology, size, location, the condition of the surrounding mucosa, and ulcer staging. Gastric ulcers are classified into three stages: active, healing, and scar stages.

### Data collection

Data were retrieved from electronic medical records and subjected to quality control; cases with incomplete data were excluded. Demographic and baseline information included sex, age, ethnicity, education, body mass index (BMI), smoking status, alcohol consumption, and family history of cancer. Major findings from MCCG included tumors and high-risk events, i.e., CAG, gastric ulcer, and gastric polyps. The serological tests included gastrin-17 (G-17), pepsinogen-I (PG-I), pepsinogen-II (PG-II), carcinoembryonic antigen (CEA), and carbohydrate antigen 19-9 (CA19-9) (29–32). At least one assessable outcome among these high-risk events was required, including CAG, gastric polyps (polypoid lesion), and gastric ulcers diagnosed by MCCG (18, 19). Mild-to-moderate and severe atrophic gastritis were defined by serostatus: (i) 20 ng/mL ≤ PG-I < 70 ng/mL and PG-I/II ratio < 3.0, and (ii) PG-I < 20 ng/mL and PG-I/II ratio < 3.0, respectively (37). High and very high levels of serum G-17 were defined as >15 pmol/L and >30 pmol/L, respectively (30). The in-house cut-off value for CEA seropositivity was ≥5 ng/mL, whereas the in-house cut-off value for CA19-9 seropositivity was ≥30 kU/L. *Hp* infection status was not assessed due to insufficient testing in this series.

### Statistics

The prevalence rates of high-risk events (per 1,000 persons) were estimated with 95% confidence intervals (CIs) based on event detection rates. For baseline characteristics, the chi-square test was used for categorical variables, and the Wilcoxon test was used for ranked variables or continuous variables, where applicable. Box plots of serologic test levels were generated. The levels of serological tests were compared between MCCG-detected event-negative and event-positive groups using the Wilcoxon rank test, with medians and interquartile ranges (IQRs) reported. Meanwhile, the severity classification of serological tests was also compared between MCCG-detected event-negative and event-positive groups. Logistic regression was used for multivariate analyses to estimate adjusted diagnostic odds ratio (aDOR), and its 95% CIs. If significant, the association strength was classified as aDOR 1.0–1.5 for weak (−), 1.5–3.0 for mild (+), 3.0–10.0 for moderate (++), and ≥10.0 for strong (+++). Sex and age classifications were adjusted in Model 1, while the classifications of ethnicity, BMI, smoking, alcohol drinking, and family history of cancer were additionally adjusted in the model 2 as sensitivity analyses (education level was not adjusted due to high proportion of unclear data). Non-parametric receiver operating characteristic curve (ROC) analysis was used to assess the capability of predicting high-risk events, while sensitivity (SEN), specificity (SPE), positive likelihood ratio (PLR), and negative likelihood ratio (NLR), and area under the curve (AUC) were estimated. The diagnostic strength was classified as AUC ≥ 0.9 for excellent (++), 0.7 ≤ AUC < 0.9 for moderate (+), and AUC < 0.7 for poor (−). All statistical tests were two-sided, and statistical significance was defined as a *p*-value < 0.05. STATA/SE 14.0 software was used for statistical analysis.

### Registration and reporting

The study protocol was reported elsewhere (ClinicalTrials.gov Identifier: NCT03597672). This cross-sectional study was reported in accordance with the Strengthening the Reporting of Observational Studies in Epidemiology (STROBE) statement (38), and the REporting of studies Conducted using Observational routinely collected data (RECORD) (39).

## Results

### Prevalence of high-risk events and risk factors

**Figure 1:**
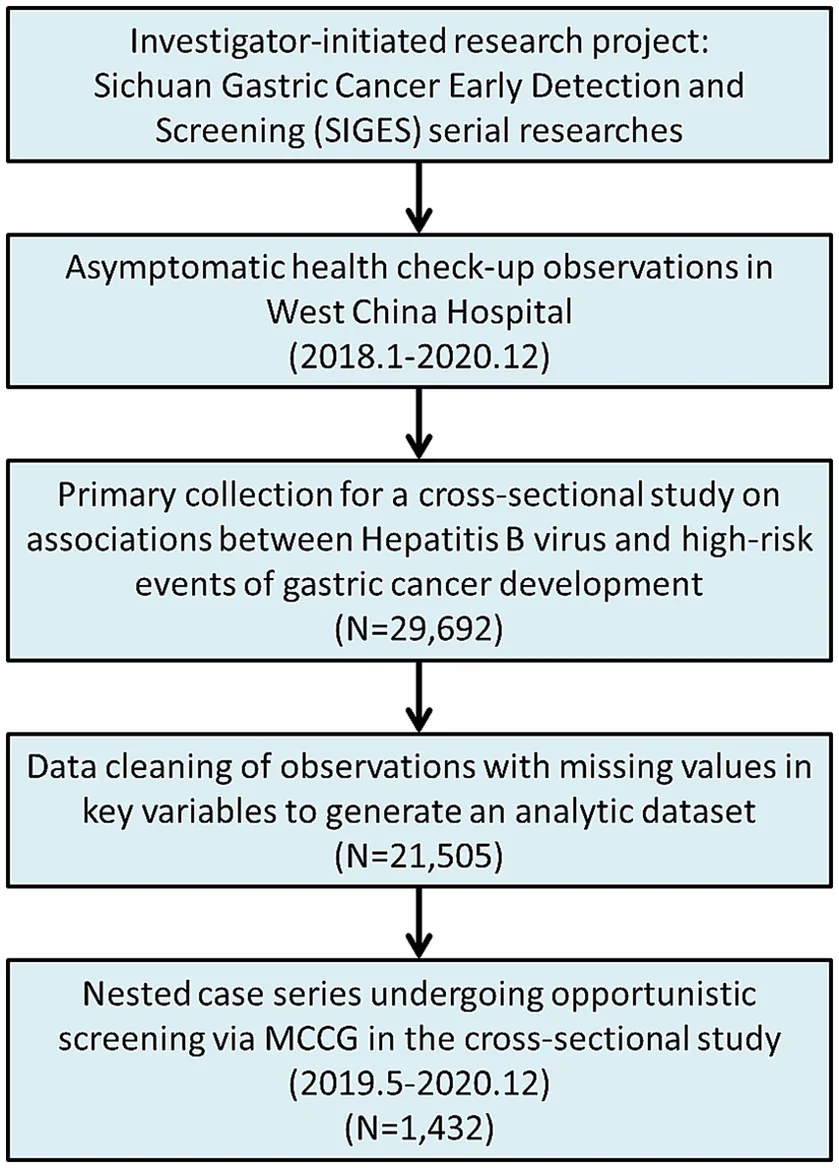
Diagram of observation collection.

**Figure 2:**
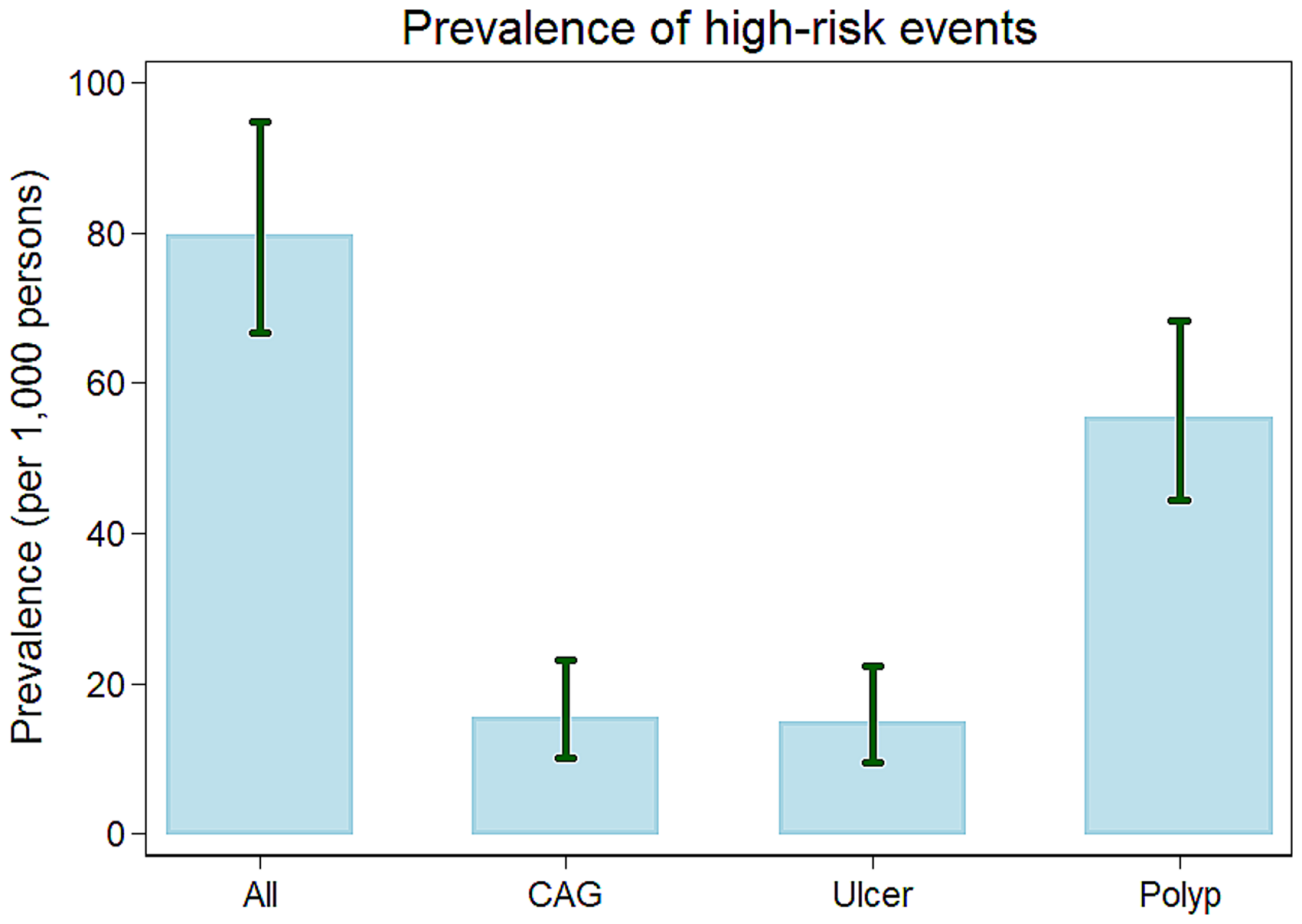
The prevalence of high-risk events for gastric cancer development by opportunistic MCCG.

A total of 1,432 eligible healthy individuals who underwent routine health check-ups were included (Figure 1). No tumors were detected in the present series, but MCCG found 114 cases with at least one high-risk event. The overall detection rate was 79.6 per 1,000 persons (95% CI 66.7–94.8 per 1,000 persons) for all high-risk events (Table 1; Figure 2). In particular, the incidences of individual events were 15.4 per 1,000 persons (95% CI 10.1–23.2 per 1,000 persons) for CAG, 14.7 per 1,000 persons (95% CI 9.6–22.4 per 1,000 persons) for gastric ulcer, and 55.2 per 1,000 persons (95% CI 44.5–68.3 per 1,000 persons) for gastric polyps, respectively (Table 1). The baseline characteristics were generally comparable between the MCCG-negative and MCCG-positive groups, with the exception of age (Table 2). Older individuals exhibited significantly higher rates of high-risk events (*p* < 0.001), particularly increased at≥60 years old.

**Table 1:** Prevalence of high-risk events of gastric cancer in the present cross-sectional study.

MCCG findings	Cases	Prevalence (95% CI) (per 1,000 persons)
All high-risk events	114	79.6 (66.7–94.8)
CAG	22	15.4 (10.1–23.2)
Ulcer	21	14.7 (9.6–22.4)
Polyps	79	55.2 (44.5–68.3)

CAG, chronic atrophic gastritis; CI, confidence interval; MCCG, magnetically controlled capsule gastroscopy.

**Table 2:** Baselines of observations between MCCG negative findings and all high-risk events groups in the present cross-sectional study.

Variables	MCCG Negative findings		All MCCG high-risk events		*p*
	*n*	(%)	*n*	(%)	
Sex					0.787
Female	872	92.18	74	7.82	
Male	446	91.77	40	8.23	
Age (yrs)					<0.001
≤0.	83	92.22	7	7.78	
≤0.7	251	95.44	12	4.56	
≤0.5	546	93.65	37	6.35	
≤0.3	330	88.71	42	11.29	
≤1.	93	91.18	9	8.82	
>70	15	68.18	7	31.82	
Ethnics					0.142
Han	1,264	92.26	106	7.74	
Minority	54	87.10	8	12.90	
Education level					0.527
<Bachelor	605	90.70	62	9.30	
≥.30 Bachelor	425	91.79	38	8.21	
Unclear	288		14		
BMI (kg/m^2^)					0.144
Normal (g/m < 25)	674	93.09	50	6.91	
Underweight (<18.5)	40	93.02	3	6.98	
Overweight (<30)	525	90.67	54	9.33	
Obese	78	91.76	7	8.24	
Unclear	1		0		
Smoking					0.254
Never	813	91.35	77	8.65	
Current or ever	495	93.05	37	6.95	
Unclear	10		0		
Alcohol drinking					0.144
Never	562	90.79	57	9.21	
Current or ever	747	92.91	57	7.09	
Unclear	9		0		
Family history of cancers					0.429
None	1,091	92.22	92	7.78	
Other malignancies	189	90.87	19	9.13	
Gastric cancer	26	89.66	3	10.34	
Unclear	12		0		

BMI, body mass index; kg, kilogram; m, meter; MCCG, magnetically controlled capsule gastroscopy; yrs, years.

### Associations between serology and MCCG findings

**Figure 3:**
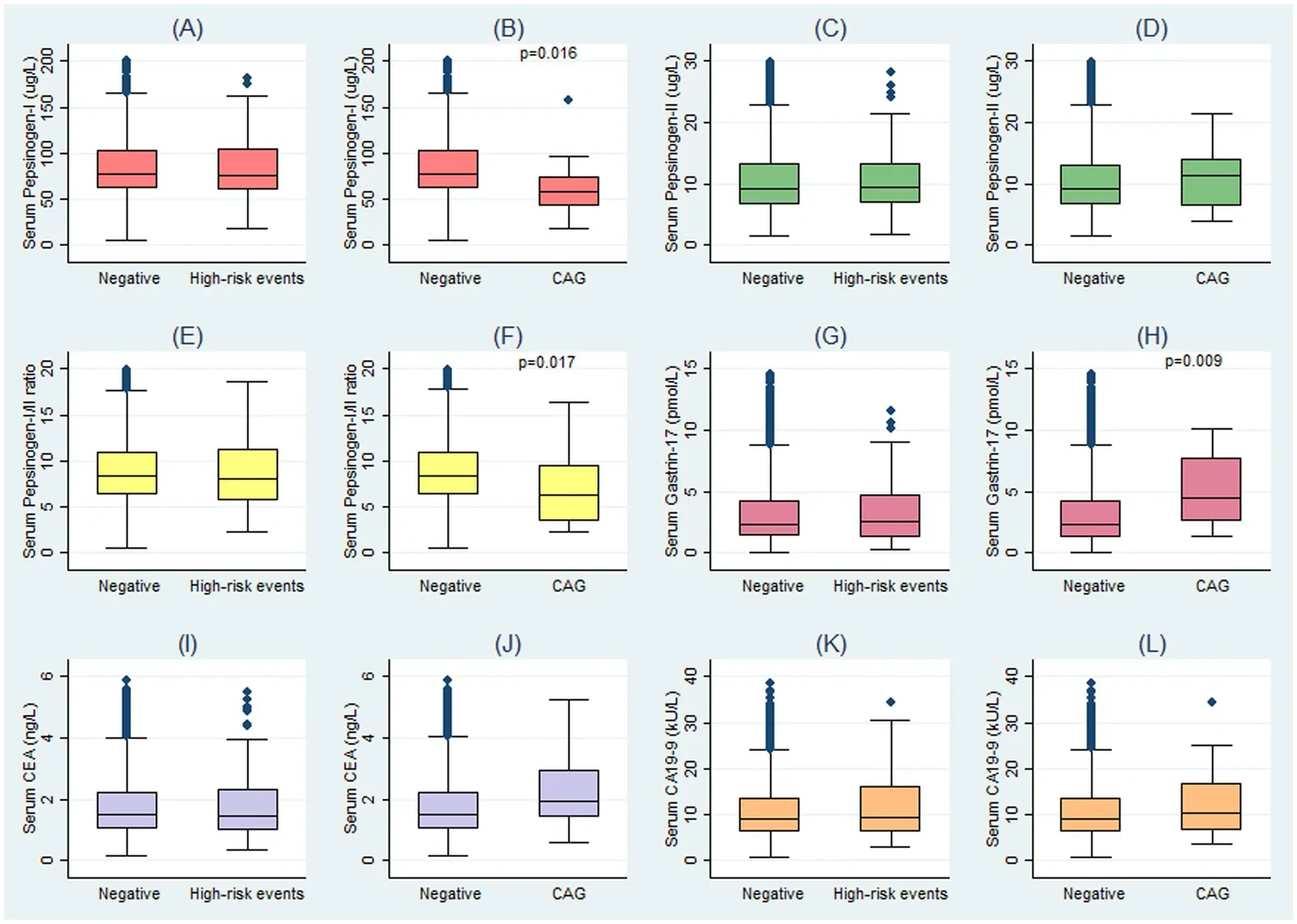
Box plots of serologic biomarker levels **(A,B)** pepsinogen I; **(C,D)** pepsinogen II; **(E,F)** pepsinogen I/II ratio; **(G,H)** gastrin-17; **(I,J)** CEA; **(K,L)** CA19-9.

There were no significant differences in the serum levels or severity classifications of the serologic tests between the MCCG-positive and MCCG-negative groups (Table 3; Table 4). However, in the MCCG-detected CAG outcome dataset, the serum levels PG-I (median = 58.9 ug/L, IQR 44.3 ug/L–85.2 ug/L, *p* = 0.016) and PGR (median = 6.2, IQR 3.4–9.5, *p* = 0.017) were significantly lower in the MCCG-detected CAG group, as well as serum G-17 higher in the MCCG-detected CAG group (median = 5.1 pmol/L, IQR 2.8 pmol/L–9.6 pmol/L, *p* = 0.009) (Table 3). Box plots of the serological test levels are presented in Figure 3. In addition, a high level of serum CEA was more frequent among MCCG-detected CAG cases (Table 4).

**Table 3:** Comparisons on levels of serologic biomarkers between event-positive and event-negative groups.

Factors	All MCCG high-risk events			MCCG CAG		
	Negative	Positive	*p*	Negative	Positive	*p*
Serum Pepsinogen-I (ng/mL)	78.6 [61.9, 105.5]	76.3 [59.8, 109.7]	0.622	78.7 [61.9, 106.1]	58.9 [44.3, 85.2]	0.016
Serum Pepsinogen-II (ng/mL)	9.2 [6.8, 13.9]	9.8 [6.9, 13.8]	0.579	9.3 [6.8, 13.9]	12.0 [6.5, 15.1]	0.508
Pepsinogen-I/II ratio	8.5 [6.3, 11.2]	8.2 [5.7, 11.3]	0.442	8.5 [6.3, 11.2]	6.2 [3.4, 9.5]	0.017
Serum Gastrin-17 (pmol/L)	2.5 [1.4, 5.1]	2.7 [1.3, 5.6]	0.832	2.5 [1.4, 5.1]	5.1 [2.8, 9.6]	0.009
Serum CEA (ng/mL)	1.5 [1.0, 2.3]	1.5 [1.0, 2.3]	0.850	1.5 [1.0, 2.3]	2.0 [1.5, 3.0]	0.095
Serum CA19-9 (kU/L)	9.1 [6.3, 13.5]	9.9 [6.4, 16.3]	0.168	9.1 [6.3, 13.6]	10.3 [6.4, 16.7]	0.534

Median and interquartile range were reported, and the Wilcoxon rank test was used.

CAG, chronic atrophic gastritis; MCCG, magnetically controlled capsule gastroscopy.

**Table 4:** Comparison of serologic biomarker stratifications between event-positive and event-negative groups.

Factors	MCCG all high-risk events			MCCG CAG		
	Positive	%	*p*	Positive	%	*p*
Serum Pepsinogen-I			0.677			0.064
Normal	66	8.15		5	0.62	
Low	42	8.00		10	1.90	
High	6	6.19		1	1.03	
Serum Pepsinogen-II			0.707			0.763
Normal	87	7.79		12	1.07	
Low	2	33.33		0	0.00	
High	25	8.09		4	1.29	
Pepsinogen-I/II ratio			0.071			<0.001
Normal	108	7.75		13	0.93	
Low	6	15.79		3	7.89	
Serum Gastrin-17			0.712			0.472
Normal	91	8.08		14	1.24	
Low	16	7.73		0	0.00	
High	6	9.52		2	3.17	
Very high	1	2.78		0	0.00	
Serum CEA			0.110			0.020
Normal	108	7.78		14	1.01	
High	6	14.63		2	4.88	
Serum CA19-9			0.305			0.202
Normal	101	7.85		12	0.93	
High	4	12.90		1	3.23	

CAG, chronic atrophic gastritis; MCCG, magnetically controlled capsule gastroscopy.

**Figure 4:**
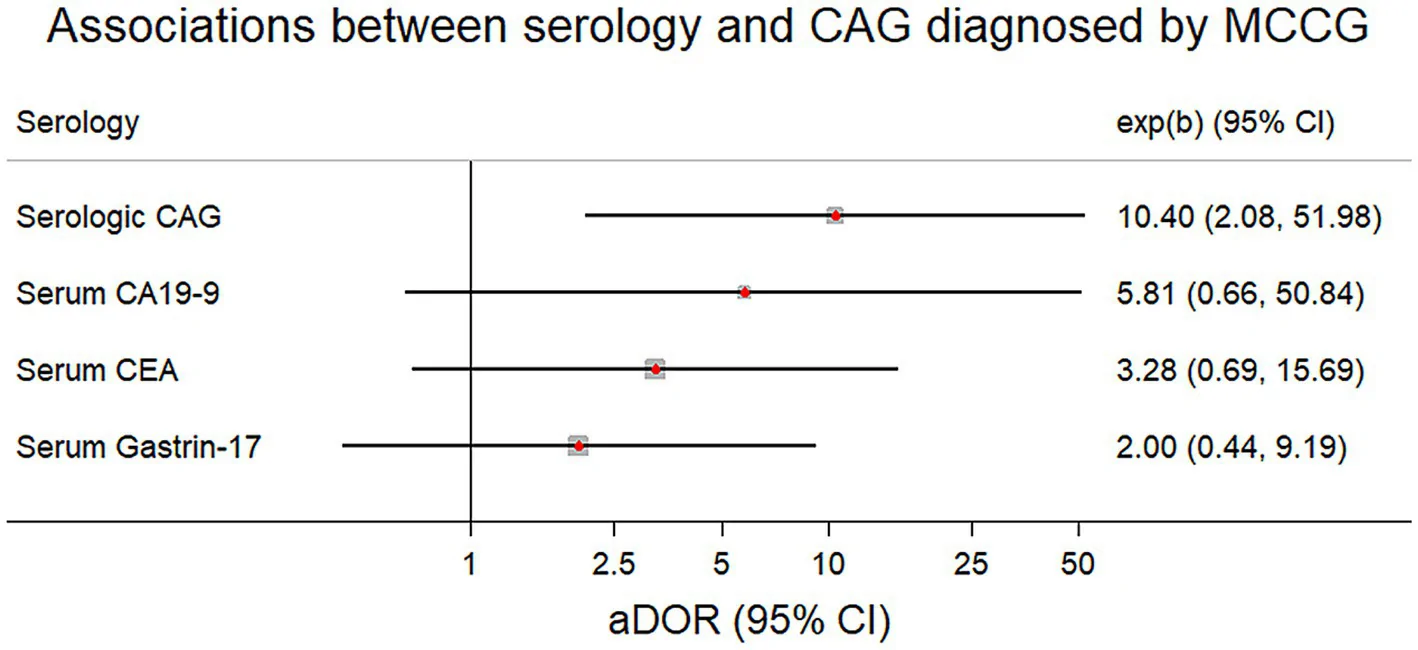
Associations between serology and MCCG-detected CAG.

In diagnostic analyses, the serologic tests demonstrated poor sensitivity across all MCCG-detected event datasets, with all the AUCs estimated at low levels (<0.600) and demonstrating low sensitivity (<15%) but high specificity (>90%) (Tables 5–8). However, serologic CAG was significantly associated with MCCG-detected CAG (aDOR = 10.40, 95% CI 2.08–51.98) (Figure 4), despite having poor diagnostic strength (AUC = 0.555, SEN = 12.5%, SPE = 98.6%) (Table 5). In addition, CA19-9 seropositivity was significantly associated with MCCG-detected gastric ulcer (aDOR = 7.86, 95% CI 1.58–39.08), also with poor diagnostic strength (AUC = 0.542, SEN = 10.5%, SPE = 97.8%) (Table 8). Seropositivity for G-17 and CEA was not associated with, nor capable of diagnosing, any high-risk event (Tables 6, 7). The sensitivity analyses of the aDOR in regression Model 2 showed consistent results (Tables 5–8).

**Table 5:** The diagnostic strength of serologic CAG associated with MCCG findings.

MCCG findings		Serologic CAG		Association, aDOR (95% CI)		Diagnostic strength				
		Negative	Positive	Model 1	Model 2	SEN	SPE	PLR	NLR	AUC (95% CI)
All high-risk events	Negative	1,299	19	1.82 (0.52–6.31)	1.77 (0.51–6.16)	2.63%	98.56%	1.83	0.99	0.506 (0.491–0.521)
	Positive	111	3							
CAG	Negative	1,396	20	10.40 (2.08–51.98)	8.62 (1.54–48.20)	12.50%	98.59%	8.85	0.89	0.555 (0.472–0.639)
	Positive	14	2							
Ulcer	Negative	1,389	22	N/A	N/A	0	98.44%	0	1.02	0.492 (0.489–0.495)
	Positive	21	0							
Polyps	Negative	1,332	21	0.79 (0.10–6.05)	0.76 (0.10–6.05)	1.27%	98.45%	0.82	1.00	0.499 (0.486–0.511)
	Positive	78	1							

aDOR, adjusted diagnostic odds ratio; AUC, area under the curve; CAG, chronic atrophic gastritis; CI, confidence interval; DOR, diagnostic odds ratio; MCCG, magnetically controlled capsule gastroscopy; N/A, not applicable; NLR, negative likelihood ratio; PLR, positive likelihood ratio; SEN, sensitivity; SPE, specificity.

**Table 6:** The diagnostic strength of serum Gastrin-17 associated with MCCG findings.

MCCG findings		Serum Gastrin-17		Association, aDOR (95% CI)		Diagnostic strength				
		Non-high	High	Model 1	Model 2	SEN	SPE	PLR	NLR	AUC (95% CI)
All high-risk events	Negative	1,226	92	0.89 (0.40–1.98)	0.90 (0.40–2.02)	6.14%	93.02%	0.88	1.01	0.496 (0.473–0.519)
	Positive	107	7							
CAG	Negative	1,319	97	2.00 (0.44–9.19)	1.78 (0.37–8.61)	12.50%	93.15%	1.82	0.94	0.528 (0.444–0.612)
	Positive	14	2							
Ulcer	Negative	1,313	98	0.69 (0.09–5.24)	0.68 (0.09–5.23)	4.76%	93.05%	0.69	1.02	0.489 (0.442–0.536)
	Positive	20	1							
Polyps	Negative	1,258	95	0.73 (0.26–2.07)	0.75 (0.26–2.12)	5.06%	92.98%	0.72	1.02	0.490 (0.465–0.515)
	Positive	75	4							

aDOR, adjusted diagnostic odds ratio; AUC, area under the curve; CAG, chronic atrophic gastritis; CI, confidence interval; DOR, diagnostic odds ratio; MCCG, magnetically controlled capsule gastroscopy; N/A, not applicable; NLR, negative likelihood ratio; PLR, positive likelihood ratio; SEN, sensitivity; SPE, specificity.

**Table 7:** The diagnostic strength of serum CEA associated with MCCG findings.

MCCG findings		Serum CEA		Association, aDOR (95% CI)		Diagnostic strength				
		Normal	High	Model 1	Model 2	SEN	SPE	PLR	NLR	AUC (95% CI)
All high–risk events	Negative	1,281	35	1.73 (0.69–4.31)	1.80 (0.70–4.61)	5.26%	97.34%	1.98	0.97	0.513 (0.492–0.534)
	Positive	108	6							
CAG	Negative	1,375	39	3.28 (0.69–15.69)	2.28 (0.42–12.38)	12.50%	97.24%	4.53	0.90	0.549 (0.465–0.633)
	Positive	14	2							
Ulcer	Negative	1,370	39	3.18 (0.70–14.47)	2.38 (0.50–11.31)	9.52%	97.23%	3.44	0.93	0.534 (0.469–0.598)
	Positive	19	2							
Polyps	Negative	1,312	39	0.73 (0.17–3.24)	0.92 (0.20–4.21)	2.53%	97.11%	0.88	1.00	0.498 (0.480–0.516)
	Positive	77	2							

aDOR, adjusted diagnostic odds ratio; AUC, area under the curve; CAG, chronic atrophic gastritis; CI, confidence interval; DOR, diagnostic odds ratio; MCCG, magnetically controlled capsule gastroscopy; N/A, not applicable; NLR, negative likelihood ratio; PLR, positive likelihood ratio; SEN, sensitivity; SPE, specificity.

**Table 8:** The diagnostic strength of serum CA19-9 associated with MCCG findings.

MCCG findings		Serum CA19-9		Association, aDOR (95% CI)		Diagnostic strength				
		Normal	High	Model 1	Model 2	SEN	SPE	PLR	NLR	AUC (95% CI)
All high-risk events	Negative	1,186	27	1.92 (0.63–5.81)	1.97 (0.64–6.10)	3.81%	97.77%	1.71	0.98	0.508 (0.489–0.527)
	Positive	101	4							
CAG	Negative	1,275	30	5.81 (0.66–50.84)	7.22 (0.69–75.09)	7.69%	97.70%	3.35	0.94	0.527 (0.451–0.602)
	Positive	12	1							
Ulcer	Negative	1,270	29	7.86 (1.58–39.08)	7.77 (1.36–44.36)	10.53%	97.77%	4.72	0.92	0.542 (0.470–0.612)
	Positive	17	2							
Polyps	Negative	1,213	30	0.48 (0.06–3.79)	0.49 (0.06–3.89)	1.33%	97.59%	0.55	1.01	0.495 (0.481–0.508)
	Positive	74	1							

aDOR, adjusted diagnostic odds ratio; AUC, area under the curve; CAG, chronic atrophic gastritis; CI, confidence interval; DOR, diagnostic odds ratio; MCCG, magnetically controlled capsule gastroscopy; N/A, not applicable; NLR, negative likelihood ratio; PLR, positive likelihood ratio; SEN, sensitivity; SPE, specificity.

## Discussion

**Figure 5:**
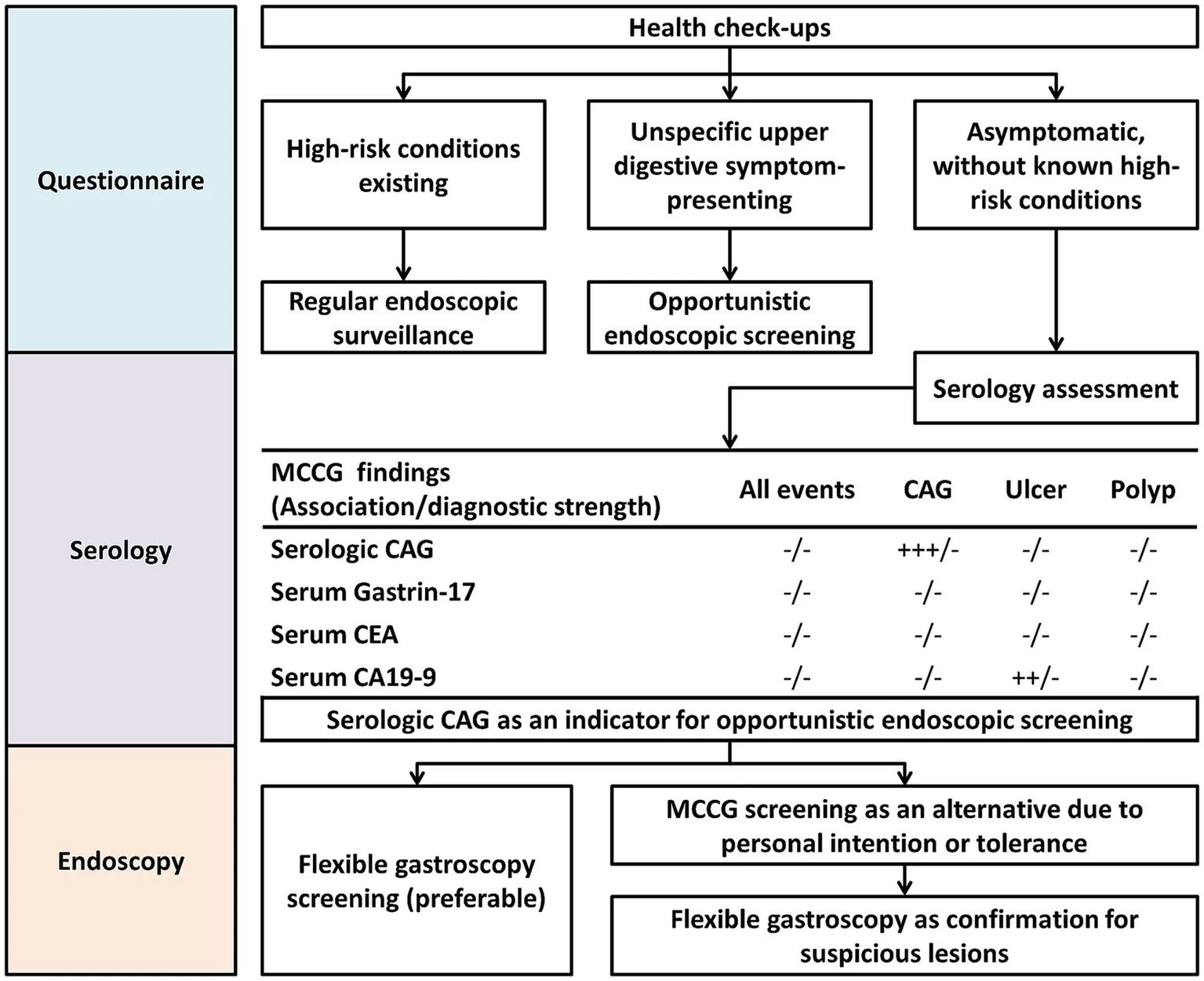
Schematic illustration of the hypothetical MCCG-integrated screening pathway for health check-up participants in the present study.

This hospital-based cross-sectional study was conducted based on the hypothesis that MCCG might exhibit acceptable detection performance, and that a sequential serology-MCCG protocol might perform well in detecting specific high-risk events in a simulated setting of opportunistic screening (Figure 5). This study found that the overall detection rate of high-risk events associated with gastric cancer by MCCG was 79.6 per 1,000 persons (95% CI, 66.7–94.8 per 1,000 persons) in total. The detection rates of CAG, gastric ulcer, and gastric polyps were 15.4 per 1,000 persons, 14.7 per 1,000 persons, and 55.2 per 1,000 persons, respectively. Serologic CAG had a significant association with MCCG-detected CAG (aDOR = 10.40, 95% CI 2.08–51.98). However, the serologic tests demonstrated poor sensitivity across all high-risk event datasets (AUC < 0.6, SEN < 15%) but exhibited high specificity (SPE > 90%).

First, the detection rates of high-risk events by MCCG appear acceptable, which indicates that MCCG may be a feasible option for opportunistic screening. In our previous study, the prevalence of mild-to-moderate CAG was estimated as 15.9 per 1,000 persons and 28.3 per 1,000 persons in healthy controls and symptomatic patients, respectively (30), which is consistent with the present result. Meanwhile, the prevalence of high-risk events increased linearly with age, particularly for CAG (30). By contrast, the prevalence of CAG is estimated to be up to 15% in U.S. populations and 25% worldwide, and even greater in specific subpopulations such as *Hp*-infected persons (40, 41). This implies that serology might underestimate the prevalence of CAG. In addition, the prevalence of gastric ulcer was estimated to be up to 5.8% in Africa, and approximately 4% worldwide though rates have been declining in high-income countries (42, 43). In the present results, the detection rate of gastric ulcer among asymptomatic individuals was relatively low at 14.7 per 1,000 persons, which indicated that substantial inter-regional disparities might exist (44). Most gastric polyps are found incidentally during esophagogastroduodenoscopy, and it is reported that sporadic gastric polypoid lesions are identified in approximately 6% of endoscopic procedures (45). Our results indicate that the detection rate (55.2 per 1,000 persons) of gastric polyps by MCCG is close to these established estimates. Therefore, MCCG is considerable as an alternative in opportunistic screening setting, especially for older individuals with less tolerance for conventional flexible endoscopy.

Second, although serology alone is still not recommended for screening for gastric cancer and its high-risk events (19), the pattern of proposed serologic testing remains meaningful to investigate. Traditionally, serum *Hp* antibody, PG-I, PG-II, and G-17 have been widely researched and utilized in health check-up, screening, and surveillance, demonstrating promising performance in the stratification of gastric cancer risk (30, 46, 47). Conventional serum tumor biomarkers such as CEA, CA19-9, CA72-4, and CA242 are associated with a significantly increased risk of gastric cancer (31, 32, 48). Although they have not yet been recommended in clinical guidelines (19), the proposed application remains interesting in further investigations. Accordingly, combining multiple serological biomarkers may be proposed to optimize the sequential serology–MCCG screening algorithm. Beyond serology, tests of breath biomarkers, gastric juice biomarkers, circulating tumor cells, long noncoding RNAs, cell-free DNA, microRNAs, and microrobots are all worthy of investigation for their potential of early detection and screening of gastric cancer and its high-risk events based on innovative technical platforms (49–51). Some pilot studies on novel blood-based miRNA diagnostic panels for gastric cancer demonstrated competent diagnostic efficacy, even in the early stages of cancer and asymptomatic cases, while their combination with existing serum biomarkers contributed to more efficient risk stratification (52–54).

Third, the sequential serology-MCCG protocol may be considered for embedding in both organized massive screening and opportunistic screening as an alternative, but further validation investigations are required (55). In the present study, all individual serological assays demonstrated excellent specificity (>90%) yet markedly low sensitivity (<15%), which accounts for the suboptimal diagnostic performance of serologic testing (AUC < 0.6). Complementary to the sequential *Hp*-endoscopy protocol (20), other serological biomarkers and their combinations are useful in early detection and screening of gastric cancer. For example, the commercial GastroPanel is validated to be a reliable triage test for identifying high-risk versus safe subgroups before endoscopy (56, 57). Similarly, our results also found pepsinogen-based identification of serologic CAG had a significantly stronger association with MCCG-detected CAG (aDOR = 10.40, SPE = 98.59%, PLR = 8.85). In addition, in the present study, tumor biomarker CA19-9 showed a significant association with gastric ulcer along with great specificity, which should be interpreted with caution. The detection rate of gastric ulcer among asymptomatic individuals was merely 14.7 per 1,000 persons. Current evidence regarding the association between serum CA19-9 and gastric ulcer remains limited and inconsistent (58, 59). Thus, we do not intend to recommend serum CA19-9 as a screening indicator under current conditions. Moreover, prospective cohort studies are proposed to longitudinally track the associations between dynamic changes in serological or other biomarkers and gastric cancer incidence, with the goal of clarifying potentially causal relationships.

On the contrary, there were several limitations that needed consideration with caution. First, in this hospital-based cross-sectional study, inter-operator bias cannot be avoided because of the endoscopist-based diagnosis. MCCG, integrated with artificial intelligence and a fully automated technique, may be helpful to improve diagnostic power in the future (28, 60). Second, in the present study, there was no case with severe atrophic gastritis diagnosed by serology. An additional observational study is suggested to focus specifically on severe CAG. Third, the major results showed poor AUCs and SENs for diagnosing high-risk events, despite some high DORs. This situation is particularly common in the analysis of rare exposure factors: when the positive rate of serologic CAG is fairly or extremely low in both the case group (2/20) and the control group (14/1,396), even if the association strength is very high, the discriminatory ability of the variable will be poor. Specifically, the small number of outcome events may yield wide CIs and impair the stability of the regression models, which reflects insufficient statistical power.

In particular, the classification of CAG depended on serology or MCCG, rather than biopsy and the pathologic gold standard (OLGA/OLGIM standards) (36). MCCG lacks biopsy capability, which may introduce misclassification bias and lead to unreliable estimates of detection rates and associations. Although the serological diagnosis of CAG has been reported to achieve an accuracy of up to 90% (61). Meanwhile, MCCG is a purely visual diagnostic tool, not a pathological one, for the diagnosis of atrophy. An observational series on MCCG in asymptomatic participants found that the detection rate of upper gastrointestinal cancers was only 0.48% confirmed by pathology, which is lower than commonly reported levels (62, 63). This limitation might critically impair the accuracy of the sensitivity and specificity estimates, which must be strongly emphasized. Therefore, the merely suggestive nature of MCCG findings must be fully acknowledged.

To improve diagnostic accuracy, we recommend that conventional flexible gastroscopy with pathological biopsy be adopted as the gold-standard reference against which MCCG findings are verified to assess the diagnostic performance of MCCG. In addition, the sequential serology–MCCG strategy we proposed was not directly assessed under the current study design; only indirect inferences regarding this two-step algorithm could be drawn. Other viable strategies consist of screening for more sensitive biomarkers or optimizing panels of existing serological markers to enhance the predictive performance of high-risk events. Therefore, compared with those without relevant findings, patients with upper gastrointestinal symptoms or local suspicious lesions identified by MCCG are more inclined to undergo conventional flexible gastroscopy promptly for further biopsy or endoscopic treatment (64). However, the present cross-sectional study lacked follow-up data, and conventional flexible gastroscopy surveillance was not performed. In future investigations, we aim to design a case-cohort follow-up. Ultimately, the confirmation of these associations should be done carefully and requires more robust investigations.

## Conclusion

MCCG has acceptable detection rates for high-risk events associated with gastric cancer according to these present exploratory findings, but is currently limited by the lack of a pathological gold reference standard. MCCG serves as an alternative for the opportunistic screening of high-risk candidates for gastric cancer to improve patient compliance, especially among older individuals. Serologic CAG was significantly associated with MCCG-detected CAG and demonstrated high specificity. Both the screening efficacy of MCCG and the cost-effectiveness of the sequential serology–MCCG protocol require further prospective investigation, with conventional endoscopy plus histopathology serving as the appropriate reference standard.

## Data Availability

The raw data supporting the conclusions of this article will be made available by the authors, upon reasonable request.
